# 

**Published:** 1880-08

**Authors:** 


### CONTENTS.

COMMUNICATIONS.

Physiology of the Salivary Glands ........ 339
Cohesive vs. Non-Cohesive Gold ........ 349
Prevention of Decay of the Teeth ........ 353
Articulating Artificial Teeth ........ 356
Report of the Kentucky State Dental Association ........ 359
Minutes of the Kentucky State Dental Society ........ 369

EDITORIAL.

Carelessness or Ignorance, which? ........ 378
Tobacco ........ 380

